# An annotation and modeling schema for prescription regimens

**DOI:** 10.1186/s13326-019-0201-9

**Published:** 2019-05-31

**Authors:** John Aberdeen, Samuel Bayer, Cheryl Clark, Meredith Keybl, David Tresner-Kirsch

**Affiliations:** The MITRE Corporation, 202 Burlington Rd, Bedford, MA 01730 USA

**Keywords:** Medication, Annotation, Prescriptions, Modeling

## Abstract

**Background:**

We introduce TranScriptML, a semantic representation schema for prescription regimens allowing various properties of prescriptions (e.g. *dose*, *frequency*, *route*) to be specified separately and applied (manually or automatically) as annotations to patient instructions. In this paper, we describe the annotation schema, the curation of a corpus of prescription instructions through a manual annotation effort, and initial experiments in modeling and automated generation of TranScriptML representations.

**Results:**

TranScriptML was developed in the process of curating a corpus of 2914 ambulatory prescriptions written within the Partners Healthcare network, and its schema is informed by the content of that corpus. We developed the representation schema as a novel set of semantic tags for prescription concept categories (e.g. *frequency*); each tag label is defined with an accompanying attribute framework in which the meaning of tagged concepts can be specified in a normalized fashion. We annotated a subset (1746) of this dataset using cross-validation and reconciliation between multiple annotators, and used Conditional Random Field machine learning and various other methods to train automated annotation models based on the manual annotations. The TranScriptML schema implementation, manual annotation, and machine learning were all performed using the MITRE Annotation Toolkit (MAT). We report that our annotation schema can be applied with varying levels of pairwise agreement, ranging from low agreement levels (0.125 F for the relatively rare REFILL tag) to high agreement levels approaching 0.9 F for some of the more frequent tags. We report similarly variable scores for modeling tag labels and spans, averaging 0.748 F-measure with balanced precision and recall. The best of our various attribute modeling methods captured most attributes with accuracy above 0.9.

**Conclusions:**

We have described an annotation schema for prescription regimens, and shown that it is possible to annotate prescription regimens at high accuracy for many tag types. We have further shown that many of these tags and attributes can be modeled at high accuracy with various techniques. By structuring the textual representation through annotation enriched with normalized values, the text can be compared against the pharmacist-entered structured data, offering an opportunity to detect and correct discrepancies.

**Electronic supplementary material:**

The online version of this article (10.1186/s13326-019-0201-9) contains supplementary material, which is available to authorized users.

## Background

Patient medication regimens are described in a variety of genres of medical documents, including prescription orders, intake interview medication lists, discharge summaries, prescribing guidelines, and medication orders. Often, at least some aspects of the regimen are described in free text; in many cases, the entire regimen is specified in free text alone. Regimen information is essential to patient care, as well as for secondary uses such as retrospective studies and pharmacovigilance, but the free text representation presents great challenges in accessing the information computationally. In this introduction we describe the availability and current state of the art of medication information extraction tools. We then describe community evaluations, open representation schemata, and corpus development efforts in the medication regimen domain.

### Medication information extraction systems

Over the last two decades, several systems have been developed to identify medication names and associated dosage attribute information in the free text of clinical reports. Early rule-based systems include CLARIT [1], MedLEE [2, 3], and MERKI [4]. MERKI is an open source system that uses a library of regular expressions and a lexicon of drug names to identify medication names and dosage attributes. Authors of this system report accuracies of 83.7% for dose, 88.0% for route of administration, and 83.2% for frequency. CLARIT, a commercial system, combines basic NLP, general and special lexicons, and pattern matching rules to identify medication names and dosage attributes. MedLEE, a commercial system developed to extract various medical concepts, identifies medication names but not dosage attributes. Additional commercial systems include LifeCode™ from A-Life Medical, Inc., Natural Language Patient Record™ from Dictaphone Corporation, and FreePharma™ from Language and Computing NV. Algorithms for these systems are not publicly available.

A 2009 assessment of the medication extraction performance of commercial systems from four vendors (Language and Computing, Coderyte, LingoLogics, and Artificial Medical Intelligence) [5] found that they did well identifying medication names (F-measure 0.932) but less well identifying attributes such as strength (F = 0.853), route (F = 0.803), and frequency (0.483), and concluded that automated extraction could support but not replace a manual process for clinical applications such as medication list generation.

The i2b2 2009 Medication Challenge shared task [6] focused on extraction of medication-related information from clinical text. The information to be extracted included medication name, dosage amount, route of administration, frequency, duration, and reason for administration. Twenty teams participated in this challenge, and while all of the top 10 systems recognized medication names well with F-measures above 0.75 F-measure, they performed less well on other attributes. The attributes that proved hardest to extract were durations and reasons, for which the highest scores were 0.525 and 0.459, respectively.

Seven of the top ten performing systems were rule-based systems [7–13]. Three of the top ten [14–16] were hybrid systems that combined machine learning and rules, including the highest ranking system [14], which used machine learning for tagging and rules for integrating related components.

PredMed [17] and MedXN [18] are two more recent systems which improve on the accuracy demonstrated by the 2009 i2b2 challenge entries. PredMed is not yet publicly available; MedXN is available as a free and open-source UIMA-based tool. Both target the same set of seven medication-related concepts, which are listed in Table 1 in comparison to other information representations.

**Table 1:** A comparison of concept coverage, and the identifiers for those concepts, in various information representations: MedXN/PredMed information extraction output, SHARPn annotation schema, FHIR clinical data structures, and TranScriptML

MedXN / PredMed	SHARPn	FHIR	TranScriptML
Dosage: amount of medication to be taken with each administration			
Dosage	Dosage	Dose	Take, Doseamount
Duration: how long patient is expected to be or has been taking the drug			
Duration	Duration		Duration
Form: Physical form of the drug			
Form	Form		Form
Frequency: how often the drug should be administered			
Frequency	Frequency	Frequency, frequencyMax, period, periodMax, periodUnits	Freq
Indication: the reason the drug is being taken by the patient			
		Reason	Indication
Medication: Name of the drug			
Medication	Medication	Medication	Medication
Miscellaneous:			
	Modifiers	Additional Instructions	Instruction
PRN: Whether the drug is to be taken as needed			
		asNeeded	PRN
Route: how the drug is administered			
Route	Route	Route	Route
Status: whether the medication is currently being taken			
	Status Change		
Strength: the amount of active drug per unit (e.g. per tablet or per ml solution)			
Strength	Strength		Strength
Timing: additional information relating to life events			
		When	Timing

Both PredMed and MedXN find spans referencing these seven concept types in text. Additionally, MedXN assigns an RxCUI id to normalize the medication name, performs coreference between medication names and regimen concepts, and attempts to assign an RxCUI normalization to the full medication concept. The full normalization produces a structured string combining the referenced regimen concepts. However, neither system normalizes the individual concepts (e.g. Frequency); individual concept references are left in their original surface text form.

### Medication annotation schemas

In the i2b2 2009 challenge, the target output included standoff annotations of six fields of medication information (medication names, doses, modes [i.e. routes], frequencies, durations, and reasons [i.e. indications]). This schema captures the text positions and surface text of each category of information, but does not capture any semantic or normalized representation for each tagged instance.

A large annotation task undertaken by Strategic Health IT Advanced Research Projects (SHARP) Research Focus Area 4 (SHARPn) consisted of annotating a variety of medical named entities in clinical notes. The annotation task was intended to support development of clinical NLP tools. The SHARPn NLP team used the annotation to improve the functionality, interoperability, and usability of a clinical NLP system, Clinical Text Analysis and Knowledge Extraction System (cTAKES), which is now publicly available as Apache cTAKES (http://ctakes.apache.org/).

The SHARPn annotation task consisted of (1) identifying mentions of clinical concepts (i.e. spans of source document text which refer to those concepts), including medications, (2) mapping them to a UMLS code [19] from the provided terminology (RxNORM for medications) [20], and (3) identifying modifiers or attributes of the mention. Terms to be annotated as a medication were terms belonging to a specified set of UMLS semantic types with RxNORM as the terminology source. In the SHARPn annotation task, the annotation was applied to a corpus of free-text clinical notes, including radiology and breast cancer notes, in which medication mentions occur primarily in sentential text or semi-structured text such as medication lists [21, 22].

SHARPn’s annotation types related to medication regimens are listed in Table 1 in comparison to other information representations; an additional annotation, Allergy_Indicator, relates to medications, but not to prescribed regimens. In addition to medication-specific attributes, several general attributes (that is, attributes not specific to a particular entity class) were applied to medication text: Negation_indicator, Uncertainty_indicator, Conditional, Subject, and Generic.

The SHARPn schema captures text positions and surface text for medication names and attributes, and normalized representation of medication names with RxNorm codes. Normalization of dosage attributes was not a focus of the annotation effort, and reasons for taking a drug (i.e., indication) were not included as part of the medication annotation task.

A 2015 BioNLP effort [23] captured annotations of medication information from Adverse Event Report documents. In addition to adverse event content, these annotations captured medication names as well as several types of regimen information: Dosage, Route, Frequency, and Duration. However, normalization of the captured information was not within the scope of this effort.

### FHIR medication resource schema

The medication information representation schema referenced above all relate specifically to inline annotation of medication regimen concepts, and the information extraction systems described have been designed and evaluated in the context of those annotation schema. To fulfil the promise of NLP-enabled downstream applications such as medication decision support and medication reconciliation, information extraction systems must produce results that are compatible with the information structures used by EHRs and other production systems. A full survey of clinical applications’ schemas for representing medication information is out of scope of this article, but TranScriptML’s attribute structure for normalizing regimen concepts was designed to be compatible with the Fast Healthcare Interoperability Resources (FHIR) standard representation.

Health Level Seven (HL7) is currently developing FHIR, a standard for RESTful exchange of clinical data [24]. FHIR is not an annotation schema and is not intended as a markup language for natural language data, but it is relevant for its inclusion of a richly detailed data structure for medication regimens in its MedicationOrder resource. FHIR MedicationOrders [25] include, among other data, regimen fields related to dosage, frequency (highly structured and allowing for normalization of expressions like “take X to Y times per Z days, with meals”), indication, and route. Table 1 shows these data types in comparison to other representations.

## Methods

### Description of the data

We developed our annotation schema and conducted our experiments using a dataset obtained from Partners Healthcare. The full dataset consisted of 2914 prescriptions, each of which included a number of fields that contain structured data (e.g., ID, medication, dose, form, frequency, duration, etc.) as well as a directions field containing unstructured text (e.g., “take 3 tablets twice a day for the next 2 weeks then stop”). Forty percent of the records were preserved as an unexamined test set for other related work, and 60% (1746 records) were used in the present study to develop and test the TranScriptML annotation schema. Our annotation effort focused exclusively on the directions field, with other fields informing the design of the annotation tag set. A simplified sample input record appears in Table 2.

**Table 2:** Sample Prescription Record

FieldName	Contents
ID	247
Medication	IBUPROFEN
Route	PO
Dose	600
Dose Units	MG
Strength	600MG
Take	1
Form	Tablet
Frequency	TID
PRN	1
PRN Reason	Pain
Duration	30
Duration Units	
Dispense Quantity	90
Dispense Quantity Units	Tablet(s)
Directions	take 2 tablets three times a day as needed for pain

The directions field was extracted from each of the training records to create 1746 short text files for annotation.

### Annotation and modeling environment

**Fig. 1:**
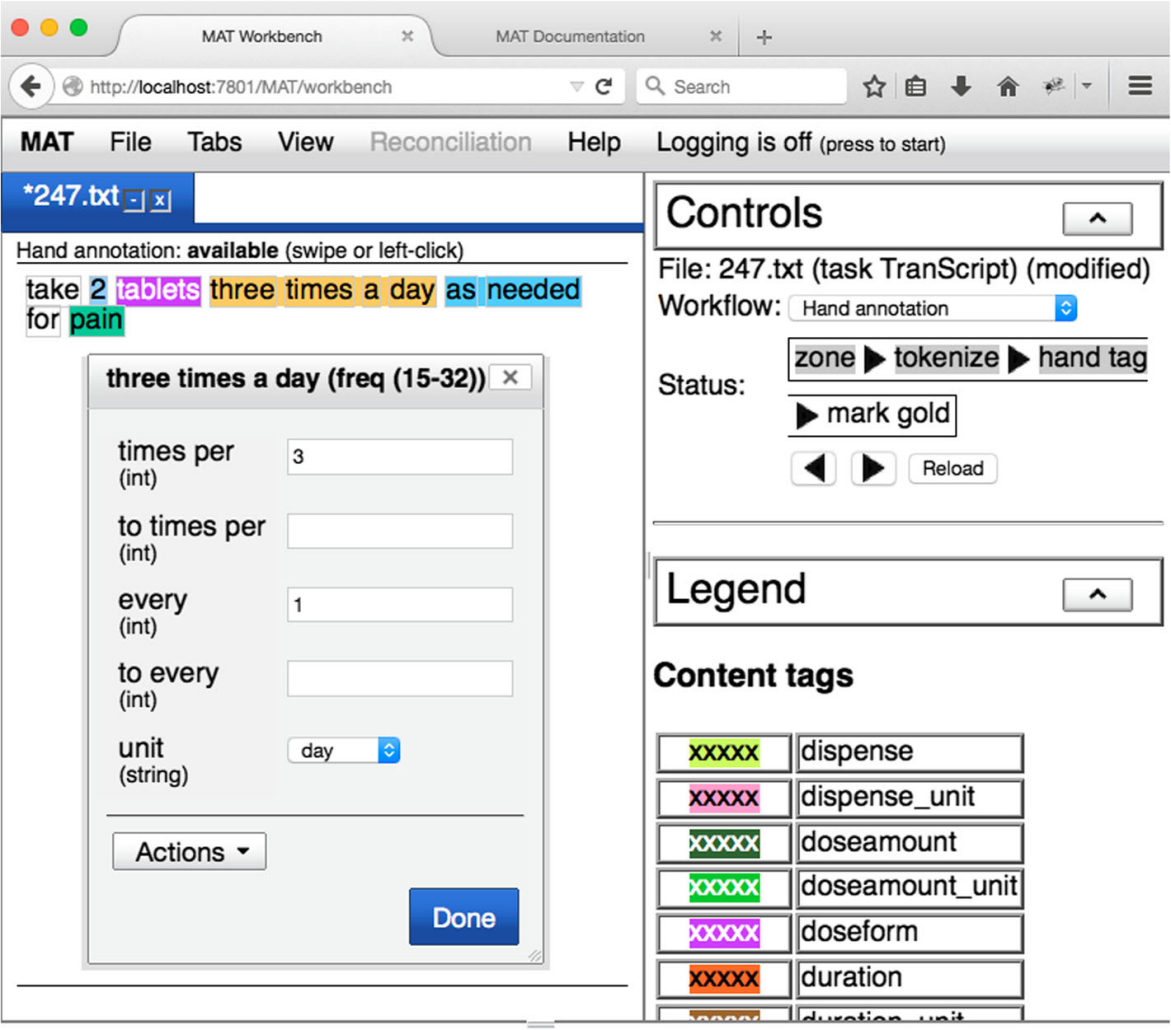
Sample record in MAT annotation environment

We constructed our annotation schema, conducted our annotation, reconciled our results, and built our models using the MITRE Annotation Toolkit (MAT). (MAT is a generalization and extension of the MIST de-identification system [26].) Open-source installation files and full documentation of MAT are available at http://mat-annotation.sourceforge.net/. MAT provides a declarative language for specifying the details of an annotation task, including tag names, attributes, and relations, as well as annotation workflows. MAT also provides a facility for building predictive models (via machine learning) from and conducting experiments with annotated data. The model building component implements machine-learning algorithms including Conditional Random Fields span annotation and Maximum Entropy classification. A sample record being annotated in MAT appears in Fig. 1.

### Annotation Schema

Throughout the remainder of the paper, we will use the following terms with specific meanings: a *tag* refers to an annotation denoting that a medication regimen concept is described in a particular part of a document; a *label* refers to the category to which the tagged concept belongs (e.g. DOSEFORM, DURATION); a *span* refers to the specific portion of the document (defined by start and end character indices) to which the tag applies; an *attribute* is an annotated property of a tag (specific to the label type) which by itself or in combination with other attributes assigns a normalized semantic representation of the tagged concept.

Our annotation schema, TranScriptML, is designed to provide flexible markup and representation of the regimens described in prescription directions. It was developed iteratively; each iteration included redundant annotation of small subsets of the corpus (~ 40 documents) by four annotators (A1-A4) using candidate TranScriptML schema versions. Discrepancies and flagged issues were discussed by all four annotators together after each iteration, which served the dual purposes of refining the schema and resolving annotator misunderstandings prior to primary annotation (which is described later). Once the schema stabilized, all documents used in schema development were reannotated along with the remainder for the sake of corpus consistency. TranScriptML contains 19 tag types, each with associated attributes. TranScriptML is a detailed representation that expands significantly on the complexity of medication regimens that can be described by the schemata used in earlier representations such as those used in the i2b2 and SHARPn medication annotation challenges. For example, TranScriptML’s attribute structure allows full specification of frequency ranges (e.g. “every 4-6 hours”), preserves the differences in meaning of frequency information that is stated as periods rather than frequencies (e.g. “every three days” vs. “three times per day”), and enables specification of additional timing information (e.g. “1 hour after meals”). The detailed attribute structure for dose, strength, frequency, and timing information is mappable to the detailed data structures used in FHIR’s MedicationOrder resource, described earlier. The list of tag descriptions appears in Table 3.

**Table 3:** TranScript annotation tag Label type descriptions

Tag	Example	Description
Dispense	120	The quantity of a medication to be issued by the pharmacist.
Dispense_unit	tablets	
Medication	Ibuprofen	Text specifying a specific pharmaceutical product.
Take	2	The quantity of medication per application, from the patient’s perspective.
Strength	400	The amount of active ingredient per physical quantity of medication.
Strength_unit	mg	
Doseamount	800	The amount of active ingredient per application of medication.
Doseamount_unit	mg	
Doseform	capsules	The form of medication taken. Often marked in conjunction with TAKE.
Duration	3	The period of time a patient should continue using a medication.
Duration_unit	weeks	
freq	3–4 times per day	The frequency of use of a medication.
Timing	before meals	The intended timing of medication use or application. Differs from FREQ in that it specifies temporal alignment of doses rather than patterns of repetition.
PRN	as needed	Text indicating medication to be taken only as needed.
Indication	pain	Condition for which medication is taken.
Route	p.o.	A medication’s manner or point of application to the body.
Refill	no refills	Indicates whether refills are allowed or not.
Sub_status	no substitutions	Indicates whether substitutions are allowed or not.
Instruction	if pain persists, call your physician	Text indicating patient actions that are not captured by another tag.

There are several tag label types represented in TranScriptML. Simple span-only tags such as PRN and INDICATION mark spans of text that refer to corresponding concepts; these tags identify the concept spans but have no additional attributes to describe and normalize the content. Other tags have attributes associated with them that encode the semantics that the text spans describe. These attributes are either numeric (e.g., quantity of a DOSEAMOUNT), text strings (e.g., units or events), or Boolean (e.g., REFILLs allowed or not allowed). Some tags are complex, with multiple attributes (e.g, FREQ and TIMING). A list of tags and their attributes appears in Table 4.

**Table 4:** Transcript annotation tag label types and their attributes

Tag	Attributes	Type (Values)
Dispense	quantity, to_quantity	Numeric
Doseamount	amount, to_amount	Numeric
Strength		
Take		
Duration	num, to_num	Numeric
Dispense_unit	unit	string (select from fixed list)
Doseamount_unit		
Strength_unit		
Duration_unit	unit	string (minute, hour, day, week, month, other)
Doseform	form	string (select from fixed list)
Medication	name	string (free text defaults to text span)
Route	route	string (select from fixed list)
	side	string (left, right, both)
Freq	times_per, to_times_per, every, to_every	numeric
	unit	string (minute, hour, day, week, month, other)
Timing	offset, to_offset	numeric
	offset_unit	string (minute, hour, day, week, month, other)
	direction	string (before, after, other)
	event	string (breakfast, lunch, dinner, meals, morning, noon, afternoon, evening, bedtime, procedure, treatment, other)
Instruction	note	string (free text input)
Refill	refill	boolean
Sub_status	subst	boolean
PRN	(no attributes)	
Indication		

### Annotation effort

Four annotators (A1-A4) participated in the study, and each document in the corpus was double-annotated. The 1746-document corpus was divided into 4 groups (G1- G4), with each annotator individually tagging all documents in 2 of the groups. After the initial annotation, each group of documents was adjudicated by a third annotator who had not been one of the primary annotators of that group. Finally, annotators A1 and A2 reconciled the entire corpus. This double-annotation, followed by the two-stage adjudication and reconciliation process was an effort to ensure we produced a consistently tagged corpus.

### Model building

We conducted several learning experiments, described below, to model the annotations in our corpus.

We used the Carafe [27] Conditional Random Fields (CRF) engine included with the MAT distribution to learn models of tag spans and labels. For span tagging and labeling, we used MAT’s default English tokenizer and the following feature set:

- Prefix and suffix ngrams of length up to 3
- Whether the current or previous token starts with a capital letter
- Whether the current token contains a digit
- The surface form of the current token
- The surface form of each of the single tokens 1, 2, or 3 tokens away from the current token

Depending on the intended use case of a trained model, the relative importance of precision and recall may not be equal, but rather there may be a particular need for high recall, or high precision. Carafe includes a parameter (prior_adjust) to adjust the tradeoff between precision and recall; we used this to build three span label models: one biased toward high recall (prior_adjust set to − 3), one biased toward high precision (prior_adjust set to + 3), and one with balanced recall and precision (default prior_adjust of 0 applied). Adjusting this parameter can result in higher recall at the expense of lower precision, or higher precision at the expense of lower recall.

For modeling the attributes of tagged spans, we used several different methods and combinations of methods, because there are several different types of attributes (numeric, string, Boolean), and many tags include multiple attributes of different types. We describe the attribute modeling methods below in the context of particular tags and classes of tags. Classifiers for modeling attributes were trained using Carafe’s Maximum Entropy engine. Model building experiments for both span annotation and attribute learning used an 80/20 training/test split of the 1746-document annotated corpus.

### Preprocess

Before modeling attributes, we normalize number expressions by using a numeric retokenizer that maps all number expressions to canonical forms. For example, *three* is mapped to *3*, *one and a half* to *1.5*, and *4* to *4*. Except where noted below, prescriptions containing normalized number expressions are the inputs for the modeling experiments.

### Frequency

The FREQ tag is complex, encoding the times-per-day and/or timing interval for medications, as well as units for these numbers. As such, it contains both numeric and string attributes, and we explored several methods for modeling the attributes. For example, “take 2-3 times per day” would involve a FREQ tag with attributes *times_per* = 2, *to_times_per* = 3, *every* = 1, *to_every* = null, and *unit* = DAY. The *Baseline* method simply counts each UNIT value and applies the most common value, along with the default numeric feature values of *times_per* = 1, *to_times_per* = null, *every* = 1, and *to_every* = null. The *Hybrid* method used a simple model containing bag of words, bigrams, and count of number expressions as features for learning the value of the UNIT attribute, as well as to determine whether the FREQ instance is an interval (every N time-units), or a count (N times per time-unit) phrase. Our *Classifier* method is a variation on the *Hybrid* method that adds a classifier for each numeric attribute, normalizing to allow only 1–12, 15, 30, 45 and 60 as valid values.

### Timing

The TIMING tag is also complex, with multiple numeric and string attributes. The *Baseline* method chooses the most common value for each attribute. The *Hybrid* method builds a classifier for *direction*, *event*, and *offset_unit*, and maps the numeric attributes directly from the normalized token list. The *Classifier* method adds a classifier for the numeric attributes as well, using the same features as used for the FREQ tag, and normalizing numeric attributes to the same list of valid values.

### Numeric attributes

DISPENSE, DOSEAMOUNT, STRENGTH, TAKE, and DURATION all have only numeric attributes. We experimented with just two conditions here, *Un-normalized*, in which the source string is mapped as-is to the attribute (e.g., “take <TAKE amt=‘three’>three</TAKE> tablets”), and *Normalized*, in which the numeric retokenizer preprocess is applied (e.g., “take <TAKE amt=‘3’>three</TAKE> tablets”).

### Choice attributes

Applying the unit tags (DISPENSE_UNIT, DURATION_UNIT, etc.) and ROUTE involves selecting attribute values from fixed lists. We explored four methods for modeling these attributes. The *Baseline* method simply chooses the most common value seen in the training data. The *Literal Match* method memorizes the mapping between text spans and attribute values in the training data, and uses the most common match. As a fallback, if a span does not appear in the training data, but matches one of the available values, the value is selected. The *Levenshtein Fallback* method is similar to the *Literal match* method, but the fallback method selects the value having the smallest edit distance [28] from the span, rather than requiring an exact match. Finally, the *Classifier* method builds a classifier for each attribute, using bag-of-words and bigrams as features.

### Boolean attributes

For the Boolean attribute tags (REFILL and SUB_STATUS), our *Baseline* method chooses the most common value seen in the training data, and the *Classifier* method builds a classifier for each attribute using bag-of-words and bigrams as features.

## Results

### Pairwise agreement

Because each document was annotated by exactly two of the four annotators, we calculated pairwise agreement for each of the four annotator pairings (G1-G4) by calculating the F-measure between each set of annotations. These calculations are presented both broken down by tag label and also in total, and they reflect the degree of agreement between human annotators without reference to automated system output. Pairwise agreement results by F-measure appear in Table 5.

**Table 5:** Pairwise agreement F-measures by annotation group and tag

Tag	G1	G2	G3	G4	Average
Dispense	0.800	0.606	0.750	0.750	0.727
Dispense_unit	0.870	0.611	0.421	0.556	0.614
Doseamount	0.786	0.845	0.779	0.841	0.813
Doseamount_unit	0.828	0.810	0.707	0.822	0.792
Doseform	0.943	0.917	0.939	0.950	0.937
Duration	0.868	0.738	0.647	0.824	0.769
Duration_unit	0.916	0.779	0.702	0.868	0.816
FREQ	0.919	0.897	0.850	0.883	0.887
Indication	0.729	0.729	0.795	0.698	0.738
Instruction	0.260	0.294	0.229	0.381	0.291
Medication	0.861	0.528	0.769	0.723	0.721
PRN	0.786	0.977	0.800	0.920	0.870
Refill	0.000	0.000	0.500	0.000	0.125
Route	0.689	0.674	0.713	0.520	0.649
Strength	0.154	0.476	0.500	0.000	0.283
Strength_unit	0.167	0.421	0.615	0.000	0.301
Sub_status	0.609	0.786	0.929	0.462	0.696
Take	0.955	0.830	0.847	0.928	0.890
Timing	0.774	0.504	0.721	0.609	0.652
Overall	0.752	0.685	0.720	0.747	0.726

Pairwise agreement by F-measure was fairly consistent between the groupings, with overall agreement ranging between 0.685 to 0.752. Inconsistent use of the STRENGTH, STRENGTH_UNIT and REFILL tags lowered their agreement levels. The agreement levels for the INSTRUCTION tag were also predictably low, as INSTRUCTION is a catch-all tag for capturing patient instructions not captured elsewhere. Most of the other tags had relatively high agreement levels.

### Label and span accuracy

Table 6 shows precision, recall, and f-measure scores for conditional random field modeling experiments for tag labels and spans. We report three experiments, one where the modeling is biased towards high precision scores, one biasing high recall scores, and a balanced run. The balanced run performs best overall, with an overall f-measure score of 0.748, and a narrow spread of precision and recall. Training with a bias towards precision boosts precision significantly (to 0.996), at the expense of recall (0.407). Surprisingly, training with a bias towards recall fails to boost recall (0.726) but does lower precision (0.651). Overall modeling results for labels and spans are encouraging, but show substantial room for improvement, particularly for the lower-frequency labels.

**Table 6:** Precision, recall, and F-measure results for labels and spans

Tag	# train	Balanced P & R			Precision Bias			Recall Bias		
		P	R	F	P	R	F	P	R	F
Dispense	46	1.000	0.333	**0.500**	1.000	0.364	0.222	1.000	0.333	**0.500**
Dispense_unit	73	0.857	0.316	0.462	1.000	0.348	0.211	1.000	0.316	**0.480**
Doseamount	221	0.900	0.614	**0.730**	1.000	0.275	0.159	1.000	0.500	0.667
Doseamount_unit	202	0.921	0.761	**0.833**	1.000	0.386	0.239	1.000	0.630	0.773
Doseform	533	0.975	0.826	**0.895**	1.000	0.769	0.625	0.981	0.736	0.841
Duration	205	1.000	0.754	**0.860**	1.000	0.659	0.492	1.000	0.590	0.742
Duration_unit	193	1.000	0.772	**0.871**	1.000	0.705	0.544	1.000	0.579	0.733
FREQ	1329	0.978	0.735	**0.840**	0.990	0.683	0.522	0.977	0.700	0.816
Indication	319	0.879	0.296	**0.443**	1.000	0.310	0.184	0.852	0.235	0.368
Instruction	4075	0.602	0.892	**0.719**	1.000	0.068	0.035	0.510	0.928	0.658
Medication	145	1.000	0.103	**0.188**	1.000	0.000	0.000	1.000	0.103	**0.188**
PRN	232	1.000	0.855	**0.922**	1.000	0.804	0.673	1.000	0.782	0.878
Refill	70	1.000	0.000	0.000	1.000	0.000	0.000	1.000	0.000	0.000
Route	591	0.947	0.423	**0.584**	1.000	0.408	0.256	0.884	0.363	0.515
Strength	33	1.000	0.000	0.000	1.000	0.000	0.000	1.000	0.000	0.000
Strength_unit	34	1.000	0.000	0.000	1.000	0.000	0.000	1.000	0.000	0.000
Sub_status	112	0.929	0.500	**0.650**	1.000	0.000	0.000	1.000	0.462	0.632
Take	774	0.952	0.855	**0.901**	0.993	0.726	0.573	0.962	0.761	0.850
Timing	1359	0.979	0.634	**0.770**	1.000	0.506	0.339	0.908	0.564	0.696
Overall	10,546	0.743	0.753	**0.748**	0.996	0.407	0.256	0.651	0.726	0.687

For each tag, the highest performing f-measure is presented in boldface

### Attribute accuracy

Table 7 shows accuracy results for modeling attribute values of various types. These experiments involved predicting attribute values for manually annotated span labels (thus there is no compounding of span prediction errors with attribute prediction errors). For choice attributes the *Levenshtein Fallback* and *Classifier* methods perform best (*side* being a notable exception where *Literal Fallback* outperforms *Levenshtein Fallback*). For the attributes of FREQ and the attributes of TIMING both the *Hybrid* and *Classifier* methods do quite well, with most accuracy scores in the 0.9–1.0 range. For the numeric attributes the *Normalized* method outperforms the *Un-normalized* method, by a large margin for some attributes. The exception is to_amt, where the *Un-normalized* method is slightly better. Finally, for Boolean attributes the *Classifier* method outperforms the *Baseline* method.

**Table 7:** Accuracy results for tag attributes

Choice Attributes	Baseline	Literal Fallback	Levenshtein Fallback	Classifier
form	0.597	0.944	0.965	0.944
route	0.339	0.667	0.738	0.887
side	0.792	0.935	0.173	0.935
unit	0.477	0.831	0.869	0.823
Attributes of FREQ	Baseline	Hybrid	Classifier	
every	0.808	0.984	0.981	
to_every	0.954	1.000	0.986	
times_per	0.570	0.927	0.900	
to_times_per	0.876	0.949	0.954	
unit	0.722	0.976	0.976	
Attributes of TIMING	Baseline	Hybrid	Classifier	
direction	0.396	0.990	0.990	
event	0.406	0.919	0.919	
offset	0.480	0.883	0.742	
to_offset	0.866	0.950	0.950	
offset_unit	0.430	0.826	0.826	
Numeric Attributes	Un-normalized	Normalized		
amt	0.160	0.927		
to_amt	0.882	0.857		
num	0.115	0.885		
to_num	0.836	0.885		
quantity	0.889	1.000		
to_quantity	1.000	1.000		
Boolean Attributes	Baseline	Classifier		
refill	0.333	1.000		
subst	0.500	0.615		

## Discussion

The results of our annotation efforts show that it is possible to create a detailed annotation schema that captures a variety of information about prescription directions in a structured way. Our pairwise agreement levels show that most of the tags in this schema can be applied in a consistent manner. The agreement levels show room for improvement, and point to the need to adjudicate a gold standard (which we did). There is always a tradeoff between the complexity of an annotation schema and the consistency with which it can be applied, as reflected in pairwise agreement numbers. The lower agreement numbers of the STRENGTH and STRENGTH_UNIT tags may be a result of their confusability with DOSEAMOUNT and DOSEAMOUNT_UNIT. These two sets of tags have clearly different uses, but capture similar information. In a complex annotation task such as ours these distinctions can become too subtle to apply consistently, and the more frequently occurring tags (DOSEAMOUNT and DOSEAMOUNT_UNIT in this comparison) can become the *default* in an annotator’s mind for particular text strings.

One of the lower performing tags in our label and span modeling is MEDICATION. Of the 26 MEDICATIONs in the test corpus, just three were correctly identified by label and span in the *Balanced* model. Nine were assigned an INSTRUCTION tag (and often a longer span) by the model, and 14 were missed entirely. This result is unsurprising. MEDICATION strings vary greatly, as do INSTRUCTION strings, and are very sparse in this corpus, as medications often appear only in the structured data and not in the patient prescription regimen string. The methods in this study relied solely on our small training set, whereas any system intended for production use should rely on a medication name vocabulary (such as RxNorm) as an additional source of information. Our attribute modeling experiments show that there are methods available to assign attributes automatically at a high level of accuracy. However, the best-performing methods differ for different attribute types. The *Classifier* methods tend to perform at or near the top for all classes of attributes, save numeric, which we did not model with any classification method.

Our study is limited in that it describes an annotation schema developed over a single corpus of prescription regimens. As there was no earlier effort to build on, development of the schema was a labor-intensive task, involving several rounds of pilot annotation and refinement of the schema. The schema has not been validated against a second corpus from a different source; this would be a valuable direction for future work.

Previous related work in de-identification has shown that the labor needed to apply a schema to a corpus can be significantly reduced by iteratively applying preliminary machine-learned models to unseen data as pre-taggers [29]. By doing this, the annotation task becomes a correction task (inspecting and correcting the output of the preliminary models), which has been shown to speed-up model and corpus development [30]. A logical next step for this work is to apply these *tag-a-little, learn-a-little* principles to bootstrap the development of an annotated prescription regimen corpus from a second source, to validate our approach.

## Conclusions

Through an annotation development effort, we have demonstrated a method for capturing structured data from prescription regimen strings, and have shown that the schema can be applied manually with high accuracy for many tag label types. We have further shown that conditional random field modeling techniques can apply tag labels to text spans with similar accuracy levels in this corpus, and that various modeling techniques can correctly set the attributes of these tags at high accuracy. Future work can address the applicability of these techniques to other corpora, and explore using tag-a-little, learn-a-little iterative model and corpus development to reduce the labor needed to create annotated corpora of prescription regimens.

The strings in our corpus are textual representations of prescription regimens, complete with errors. By structuring the textual representation through annotation, the text can be compared against the pharmacist-entered structured data (through one-to-one data structure mapping in the case of FHIR-compliant pharmacy data), offering an opportunity to detect and correct discrepancies.

TranScriptML is a richer representation of medication regimen information than those used in previous natural language annotation efforts, and is consistent with emerging standards for representation of structured data in the same domain. We hope these standards will encourage compatibility between clinical NLP tools and the Electronic Health Record (EHR) software ecosystem. For these reasons, we are releasing our annotation schema and guidelines alongside this report, and urge that TranScriptML or compatible representations be used in future corpus development.

## Additional files

Additional file 1: TranScriptMLAnnotationGuidelines.docx (MS Word document). TranscriptML Annotation Guidelines. Guidelines for applying the TranscriptML annotation schema to textual prescription regimens. (DOCX 56 kb)

Additional file 2: TranscriptAnnotationTask.xml (XML file). TranscriptML Task Definition. XML file defining the Transcript task for use with the MITRE Annotation Toolkit. (XML 19 kb)
