# Supplementary material for: An annotation and modeling schema for prescription regimens
Source: J Biomed Semantics. 2019 May 31;10:10. doi: 10.1186/s13326-019-0201-9 (PMC6544933; doi:10.1186/s13326-019-0201-9)
Supplement: Supplementary file 1 — TranScriptMLAnnotationGuidelines.docx (MS Word document). TranscriptML Annotation Guidelines. Guidelines for applying the TranscriptML annotation schema to textual prescription regimens. (DOCX 56 kb) [file 13326_2019_201_MOESM1_ESM.docx]

TranScriptML: Annotation Guidelines

# 1 General

These guidelines describe a framework for the annotation of concepts within pharmaceutical prescription text. A task schema compatible with the guidelines has been implemented for the MITRE Annotation Tool.

Most of the instructive content of this document relates to properties and guidelines for individual tag types, and is found in Section 2. The present section, however, contains information common to multiple tag types.

## 1.1 Conventions

Annotations in MAT are abstract, standoff annotations which can be exported in various representations. Since the native representation is not easily humanreadable, examples in this document are written as inline XML:

(1) This example has a *<*tag name attribute=‘‘attribute value’’*>*tag*<*/tag name*>* in it.

Example tags and references to the names of tags and attributes will always appear in monospace font.

## 1.2 Attributes

Each attribute of any tag is assigned one of the following value types: boolean, integer, float (decimal), or string. Some string attributes are free-text entry; others (most) have a restricted list of values to choose from, generally including a catchall value of *other*.

## 1.3 Extent

Tags should observe a principle of minimal extent: they should cover only the extent of text that communicates semantic information relevant to the tag (therefore excluding peripheral function words). For example, 2 shows a correct tag extent, while 3 and 4 show incorrect extents:

1. Take *<*freq*>*every day*<*/freq*>*
2. **<*freq>Take every day*<*/freq*>*
3. *Take every *<*freq>day*<*/freq*>*

## 1.4 Numeric attributes

Quantities should all be normalized to integers or decimals, e.g. “one” becomes 1, and “1 1/2” becomes 1.5.

## 1.5 Range attributes

Some tags have pairs of attributes allowing the specification of either a single numeric value or a numeric range (values may be either float or integer depending on the tag). These paired attributes can be recognized by naming convention; they will fit the pattern (X, to X). When annotating a single value, only the first attribute (X) should be used:

1. Take *<*take amt=6*>*6*<*/take*>*.

When annotating a range, the attributes should be used as lower and upper bounds, respectively:

1. Take *<*take amt=3 to_amt=4*>*3-4*<*/take*>*.
2. Take *<*take amt=0 to_amt=4*>*up to 4*<*/take*>*.

# 2 Tags

The TranScript annotation schema contains the following tags:

| - dispense - dispense unit - medication - take - strength - strength unit - doseamount | - doseamount unit - doseform - duration - duration unit - freq - prn - indication | - instruction - refill - route - sub status - timing - wuzza |
| --- | --- | --- |

## 2.1 dispense and dispense unit

Text indicating instructions for how much of a medication should be issued by the pharmacist should be marked using dispense (one attribute: quantity [integer]) and dispense unit (one attribute: unit [string with restricted value list]) tags.

1. please dispense *<*dispense quantity=‘‘4’’*>*4*<*/dispense*>*

*<*dispense unit unit=‘‘bottle’’*>*bottles*<*/dispense unit*>*

1. *<*dispense quantity=‘‘3’’*>*3*<*/dispense*><*dispense unit unit=‘‘month supply’’*>*month supply*<*/dispense unit*>* given.

## 2.2 medication

Use the medication tag to mark regions of text specifying a particular pharmaceutical product. This tag has one attribute (name [string]), which will prefill with the text of the span. Only change this text if there is clearly a misspelling in the name.

## 2.3 take

The take tag indicates a quantity of medication per application, from the perspective of the patient’s physical action. This differs from doseamount, which indicates the quantity of active ingredients prescribed (see 2.5). take is most often used in conjunction with doseform:

(10) Take *<*take amt=3 to_amt=3*>*3*<*/take*><*doseform form=‘‘tablet’’*>*tabs*<*/doseform*>*.

take has two attributes: amt and to_amt [float, float] (see section 1.5 on range attributes).

## 2.4 strength and strength unit

The strength and strength unit tags are used to mark the amount of active ingredient per physical quantity of medication. strength has one attribute, amt [float], and strength unit has one as well, unit [string]. A strength may be the milligram content per tablet or the concentration of a liquid or suspension:

(11) *<*strength amt=2.5*>*2.5*<*/strength*><*strength unit unit=‘‘percent’’*>*%*<*/strength unit*>* ointment.

## 2.5 doseamount and doseamount unit

Use doseamount and doseamount unit to mark the quantity of active ingredients prescribed per application of a medication. Note the difference between doseamount and take (see section 2.3).

(12) Take *<*doseamount amt=30*>*30*<*/doseamount*><*doseamount unit unit=‘‘milligrams’’*>*MG*<*/doseamount unit*>* with food.

## 2.6 doseform

Mark text indicating the form of a medication using the doseform tag with one attribute (form), a string with a fixed list of value choices. This is often in conjunction with take. See example 10.

## 2.7 duration and duration unit

Use duration and duration unit tags to mark text indicating how long the patient should continue using a medication. duration uses paired numeric attributes num and to num to allow the specification of ranges when needed (see section 1.5).

(13) use 21 mg patches for *<*duration num=‘‘1’’*>*one*<*/duration*> <*duration unit unit=‘‘month’’*>*month*<*/duration unit*>*.

Note that some phrases that superficially appear to refer to duration are actually dispense instructions (see example 9).

## 2.8 freq

Mark text indicating the frequency of use of a medication using the freq tag. It has five attributes:

- unit; The unit of time in which the frequency is expressed. Values:

day week month other

- times per and to times per; Integer values: how many (or what range of) times should the medication be used during each timespan?
- every and to every; Integer values: how many (or what range of) timespans should occur between medication uses?

In most cases, one of the two primary integer attributes should have a value of 1. Examples:

1. Take *<*freq times per=‘‘1’’ every=‘‘1’’ unit=‘‘day’’*>*daily*<*/freq*>*.
2. Apply *<*freq times per=‘‘1’’ every=‘‘2’’ unit=‘‘week’’*>*every other week*<*/freq*>*.
3. *<*freq times per=‘‘0’’ to times per=‘‘3’’ every=‘‘1’’ unit=‘‘day’’*>*up to 3x a day*<*/freq*>*.

However, in rare case, both fields will be greater than one:

1. *<*freq times per=‘‘2’’ every=‘‘3’’ unit=‘‘day’’*>*Twice every third day*<*/freq*>*.

## 2.9 timing

The timing tag is used to indicate instructions of the intended timing of a medication. This is different from the freq tag in that it relates to the alignment of individual doses rather than to patterns of repetition. Instructions should only be tagged as timing if they relate to clocks or calendars, e.g. *at noon*, *with meals*, *on the third day*. In contrast, timing of doses relating to the internal state of the patient (e.g. *at headache onset*, *during menstruation*) should instead be annotated with the instruction tag (see section 2.11). timing has four attributes:

- event; The calendar time or event referenced in the instructions.
- offset and offset unit; If applicable, how long before or after the event the medication should be used.
- direction; Whether the offset is to a time before or after the reference event.

## 2.10 prn

The prn marks language indicating that a medication should only be taken as needed. It has no attributes. See example 18.

### 2.11 indication

The indication tag is intended for text describing the condition for which a medication is being taken. This often used in conjunction with the prn tag.

(18) Take *<*prn*>*as needed*<*/prn*>* for *<*indication*>*dizziness*<*/indication*>*.

### 2.12 route

Use the route tag to mark language describing a medication’s manner or point of application to the body. It has one string attribute (route) constrained to the following values:

- mouth • inhale • teeth
- sublingual • nebulizer
- swish & swallow
- eyes • intravenous
- ears • intramuscular • swish & spit
- nasal • intradermal
- gums
- rectal • subcutaneous
- vaginal • topical • other

route also has an optional side attribute (with values *right*, *left*, or *both*)

to be used when the route refers to body parts such as eyes or ears.

### 2.13 refill and sub status

These tags indicate instructions allowing or disallowing refills and substitutions, respectively. Each has a boolean attribute denoting polarity. It should be used only for instructions relating to refillability or substitutability, not simple statements of fact about the current prescription:

1. *<*refill refill=‘‘0’’*>*No refills*<*/refill*>*, *<*sub status subst=‘‘1’’*>*generics ok*<*/sub status*>*.
2. This is a refill, substituting for zoloft.

### 2.14 instruction

instruction is a catchall tag that indicates intended patient actions that do not fall into another category.

1. Take *<*instruction*>*with food*<*/instruction*>*.
2. inject, then *<*instruction*>*call 911*<*/instruction*>*.

Miscellaneous instructions to the *pharmacist* (as opposed to the patient) should *not* be tagged.

### 2.15 wuzza

wuzza is a special tag, in that it is not intended to be a part of the target gold standard data schema. Use it to mark sections that are particularly confusing and require further discussion.
